# Bark beetles and pinhole borers (Curculionidae, Scolytinae, Platypodinae) alien to Europe

**DOI:** 10.3897/zookeys.56.529

**Published:** 2010-09-17

**Authors:** Lawrence R. Kirkendall, Massimo Faccoli

**Affiliations:** 1 Department of Biology, University of Bergen, P.O. Box 7803, N-5006 Bergen, Norway; 2 Department of Environmental Agronomy and Crop Productions – Entomology, Viale dell’Università, 16 - 35020 Legnaro (PD), Italy

**Keywords:** Invasive species, polyphagy, inbreeding, Ambrosiodmus, Ambrosiophilus, Coccotrypes, Cyclorhipidion, Dactylotrypes, Dryocoetes, Gnathotrichus, Hypocryphalus, Hypothenemus, Phloeosinus, Phloeotribus, Megaplatypus, Monarthrum, Xyleborinus, Xyleborus, Xylosandrus

## Abstract

Invasive bark beetles are posing a major threat to forest resources around the world. DAISIE’s web-based and printed databases of invasive species in Europe provide an incomplete and misleading picture of the alien scolytines and platypodines. We present a review of the alien bark beetle fauna of Europe based on primary literature through 2009. We find that there are 18 Scolytinae and one Platypodinae species apparently established in Europe, from 14 different genera. Seventeen species are naturalized. We argue that Trypodendron laeve, commonly considered alien in Europe, is a native species; conversely, we hypothesize that Xyleborus pfeilii, which has always been treated as indigenous, is an alien species from Asia. We also point out the possibility that the Asian larch bark beetle Ips subelongatus is established in European Russia. We show that there has been a marked acceleration in the rate of new introductions to Europe, as is also happening in North America: seven alien species were first recorded in the last decade.

We present information on the biology, origins, and distributions of the alien species. All but four are polyphagous, and 11 are inbreeders: two traits which increase invasiveness. Eleven species are native to Asia, six to the Americas, and one is from the Canary Islands. The Mediterranean is especially favorable for invasives, hosting a large proportion of the aliens (9/19). Italy, France and Spain have the largest numbers of alien species (14, 10 and 7, respectively). We point out that the low numbers for at least some countries is likely due to under-reporting.

Finally, we discuss the difficulties associated with identifying newly invasive species. Lack of good illustrations and keys hinder identification, particularly for species coming from Asia and Oceania.

## Introduction

The great British ecologist Charles Elton presciently referred to the effect of invasive species as “one of the great historic convulsions in the world’s fauna and flora” (Elton 1958). Enormous damage is done by nonindigenous species to ecosystems and economies (e.g. Vitousek et al. 1997, Pimentel et al. 2005, Colautti et al. 2006, Asner et al. 2008), and introduced species are considered the biggest threat to biodiversity after habitat destruction (Wilson 1992). Though the ecological and economic effects of many immigrant species are minor, some immigrant species can significantly impact the functional properties of ecosystems, disrupt food webs, displace indigenous species, or threaten food and water supplies (Kenis et al. 2009). In some cases, it is the activities of the organism itself which have these effects, but in others, such as Dutch Elm Disease, it is the microorganisms they bear in or on them (e.g. Humble and Allen 2006).

Introduced wood-borers are a major concern to regions with significant forest resources. Around the world, dozens if not hundreds of alien phytophagous insects become established every decade, and wood-borers make up a significant proportion of these (Haack 2001, 2006, Work et al. 2005, Mattson et al. 2007). Steve Wood first drew attention to the accelerating rate of introductions of bark beetles and pinhole borers (Curculionidae: Scolytinae, Platypodinae) starting with a brief article in 1977 and subsequently re-visited the topic in each of his major synoptic works (Wood, 1977, Wood 1982, Wood and Bright 1992, Wood 2007). Supplements to the world catalog also express worries over the rapidly increasing list of established alien species (Bright and Skidmore 1997, 2002). In 1995, concerned about the growing problems with identifying exotic bark beetles, Robert Haack (USDA Forest Service) and European and Asian plant protection specialists finally convinced Steve (then six years retired!) to commence work on what would be his last great achievement, the monograph of the Scolytinae of South America (Wood 2007).

There has been over three decades of discussion of the problems posed by introduced bark beetles. Steve Wood’s 1977 paper was developed from a talk given at the XIVth International Congress of Entomology in 1972. Both this and the subsequent treatment of the topic in the introductory material of the 1982 monograph (pages 25–27) were from an American point of view: which species have been introduced to the Americas, and which North, Central or South American species have become established in Europe.

With respect to exotic wood-boring insects, for North America, much is known about which invasive species are present and where (Haack 2001, 2006). We know much less about the numbers and distributions of alien species in Europe. In Wood’s 1982 treatment, only Gnathotrichus materiarius had been introduced from the Nearctic to Europe, and a recent paper (Mattson et al. 2007) operated with only five species – less than a third of the total which we report here. There are no previous reviews on the topic, and databases which have been established specifically to inform the public and policy makers about alien species in Europe are riddled with errors and incomplete (at least with regard to Scolytinae and Platypodinae).

There are two sources of newcomers to a fauna: species originally from distant regions or other continents, and those from the same region or continent which are expanding or shifting their ranges. We consider here only established species immigrant to continental Europe. Within-Europe range expansions are of interest in themselves, but ecologically and evolutionarily are a distinct phenomenon from that of the establishment of truly exotic species. We will use the term ‘alien species’ here in the sense of alien to Europe, originating outside the bounds of continental Europe.

## Methods

### Terminology

The terminology of invasion biology is much disputed (e.g. Frank and McCoy 1990, Colautti and MacIsaac 2004, and their references), so we find it prudent to define ours. We use the terms exotic, alien, and non-indigenous interchangeably, to refer to species whose native distributions lie outside of continental Europe, our reference point. We use invasive broadly to refer to alien species which have established self-sustaining populations, irregardless of whether in natural or man-made habitats; we do not use it in the restricted sense of species having known ecological or economic effects. Introduced is sometimes used to refer to deliberate introductions (Frank and McCoy 1990), but we use it more broadly to indicate spread by human-mediated transport (regardless of intent), and we use immigrant and the collective term adventive synonymously. Naturalized refers to aliens with free-living, self-sustaining populations.

While we adopt the same definition of Europe used in DAISIE and Fauna Europaea, we exclude the Macaronesian islands, preferring to focus on continental Europe (including Ireland and the United Kingdom). Consequently, we consider the Canary Island endemic Dactylotrypes longicollis to be alien to Europe, and we do not treat the alien species found on the Azores (Bright 1987) but not elsewhere in Europe.

For brevity, in taxonomic contexts, we use bark beetle to include both Scolytinae and the closely related Platypodinae. Ambrosia beetles cultivate symbiotic fungi on the walls of their tunnel systems, which fungi are the sole food of larvae and adults. All Platypodinae are ambrosia beetles, as are many genera of Scolytinae.

### Sources of data

Our starting point for listing alien bark beetles was the European database for alien organisms DAISIE (Delivering Alien Invasive Species Inventories for Europe). The DAISIE project encompasses over 11,000 species of all types of organisms, and is meant to be a central clearing house for information on biological invasions in Europe, and the database is continually updated. The geographic and taxonomic information in DAISIE is intended to play a key role in future national and international efforts to monitor and combat the spread of harmful non-native organisms. This information comes in two forms, the web-based database (DAISIE 2009a) and in lists in the recently published handbook of alien species (DAISIE 2009b).

In addition to the DAISIE website, we consulted NOBANIS (The North European and Baltic Network on Invasive Alien Species), a “Gateway to information on invasive alien species in North and Central Europe”. For further distributional data on scolytine and platypodine beetles in Europe we employed Fauna Europaea (Knížek 2004), the definitive database for scientific names of animals in Europe (native and non-native). These are the primary online resources available to the public, and likely the primary sources of information on European alien bark beetles outside of the scientific literature.

We also searched ISI Web of KnowledgeSM (and Internet more generally), but quickly found that almost none of the literature on alien bark beetles can be found by searching the web. The sources for the data in DAISIE are not given. To investigate the validity of the records available in the online databases, we searched the literature at our disposal, including the world catalog for bark beetles (Wood and Bright 1992), general works on the bark beetle fauna of Europe, country treatments, and papers with individual species records. We also availed ourselves of the generous advice and information from colleagues throughout Europe (see Acknowledgments), and of personal knowledge.

### Treatments of data

We have attempted to classify the phase of establishment of each species (Table 1), given the collection localities and dates which are available in the literature. Phases range from Phase 1 (newly collected or intercepted, no evidence of establishment) to Phase 5 (apparently distributed throughout currently suitable habitat in Europe). (Since this paper focuses on aliens for which there is evidence of establishment, we do not treat species which are in Phase 1.) We did not feel that enough was known about alien bark beetle populations (in particular, about local abundances) to apply the Stages system of Colautti and MacIsaac (2004), but acknowledge its value.

**Table 1.** The population phases which we apply to alien species in Europe.

Stage	Population level in Europe	Examples of evidence (not exhaustive)
Phase 1	Interception, recently arrived (no evidence of establishment)	Collected from imported plant material; trapped at port or near imported logs; unique, old literature records
Phase 2	local colony persisting	One area: many specimens; repeated collections; collections in natural forests far from ports of entry
Phase 3	>1 colony, not spreading.	Disjunct populations, but no sign of expanding
Phase 4	more than one large colony, spreading	Disjunct populations: Well established in several areas and still spreading
Phase 5	established throughout suitable habitats	Distributed throughout region with currently suitable climate and host plants

### Problems with data quality

As we quickly discovered to our dismay, literature documenting the discovery and spread of alien species is scattered and mostly published in obscure and difficult to obtain journals and newsletters, in a bewildering variety of languages: few of these publications are peer reviewed and almost none indexed in ISI Web of KnowledgeSM. Much of the knowledge of new discoveries seems to have been transmitted by word of mouth, in Europe.

Adding to the confusion is the fact that old names die hard. Much of the literature on introduced species promulgates names used in the original papers but which are no longer used. This is especially true of review papers and invasive species databases.

Many articles lack information on who identified the specimen(s) and what criteria were used. New locality records (even country records) seem to occasionally be based on similarity with a species which is known to be in nearby countries, or based on old, incomplete keys; both methods can easily lead to mistakes in difficult taxa, such as Hypothenemus or Coccotrypes, which only experienced specialists can identify with any degree of confidence. Almost never is information on the deposition of voucher specimens stated; to confirm the identity of the species, one must try to find and contact an author in order to locate specimens.

## Results and discussion

### Which alien species are established in Europe?

#### The species present.

There are 19 alien species established in continental Europe, according to our sources (Table 2). One of these, Megaplatypus mutatus, is a platypodine; the remainder are scolytines. Of these 19, we classify 14 as potentially expanding (Phases 2 – 4), 5 as probably currently spreading (Phases 3 – 4). All but one are considered naturalized: Xylosandrus morigerus is not known to have established populations in the wild, but seems to have a permanent presence in European greenhouses.

**Table 2.** The alien Scolytinae and Platypodinae of Europe, and the countries in which they are established. First: first record or first publication. Phase: phase of colonization, see Table 1.

Species	Established in countries	First	Phase	Notes, References
*Ambrosiodmus rubricollis (Eichhoff)	IT	2008	2	Faccoli et al. 2009.
*Ambrosiophilus atratus (Eichhoff)	IT	2007	2	Faccoli 2008, locally established.
Coccotrypes dactyliperda (Fabricius)	ES, FR, GR, HU (cultivated palms), IT, MA	1884	5	First mention is Eichhoff 1878 and 1881, also in Reitter 1913: from shops with imported dates and betelnut—no mention of established populations in Europe. ES, Garcia-Tejero 1955, definitely well established along coast. FR, Balachowsky 1949, common along coast. GR, Vasilaina-Alexopoulou et al. 1986, established. HU, György and Podlussány 2005, apparently in cultivated palms. IT, Targioni-Tozzetti 1884, established in Tuscany (earliest European record). MA, Mifsud and Knížek 2009. This species is widespread in N Africa.
*Cyclorhipidion bodoanum (Reitter)	BE, CH, DE, FR, IT, NE	1960	4	BE, Henin and Nageleisen 2005. DE, CH, Köhler 1992. DE, FR, Schott and Callot 1994, Bense and Schott 1995, Schott 2004 (as Xyleborus peregrinus). First record Alsace, 1960. IT, Audisio et al. 2008. NE, Vorst et al. 2008. AT: Knížek 2004 and DAISIE. But according to Hannes Krehan, Austrian Inst. for Forest Protection, there are no official records in AT.
Dactylotrypes longicollis (Wollaston)	CA, ES, FR, IT	1949	4	ES, Lombardero and Novoa 1994. FR, Balachowsky 1949, date seeds intercepted in New York, originating in “France”; Bovey (1987), 1^st^ France record 1955. IT, Sampò and Olmi 1975. CA, Whitehead et al. 2000. Spreading in Mediterranean, where it is probably currently confused with Coccotrypes dactyliperda.
*Dryocoetes himalayensis Strohmeyer	CH, FR	2009	4	Knížek, unpub., CH and FR, established.
Gnathotrichus materiarius (Fitch)	BE, CH, CZ, DE, ES, FI, FR, IT, NE, SE	1933	5	BE, Moucheron and Warzee 2006; CH, von Hirschheydt 1992 (1984). CZ, Knížek 2009. DE, Schedl 1966; Gladitsch 1969 (1964). ES, established, López et al. 2007 (2003). FI, Valkama et al. 1997 (1996). FR, Balachowsky 1949 (1933 was 1st Eur record). IT, Faccoli 1998 (1998). NE, Schedl 1966; Doom 1967 (1965). SE, Gillerfors 1988.
*Hypocryphalus scabricollis (Eichhoff)	MA	1991		MA, Mifsud and Knížek 2009, in ornamental Ficus.
*Hypothenemus eruditus Westwood	ES, FR, IT, MA	1924	5	Eichhoff 1878, 1881, no mention of European populations. ES, Garcia-Tejero 1955, established. FR, Balachowsky 1949 (not widespread, then). IT, Ragusa 1924. MA, Mifsud and Knížek 2009. Balachowsky 1949 says it is in ES, IT. Pfeffer 1995, throughout the Mediterranean.
Megaplatypus mutatus (Chapuis) ^a^	IT	2000	2	IT, Tremblay et al. 2000. Kills poplars.
*Monarthrum mali (Fitch)	IT	2008	2	IT, Kirkendall et al. 2008. Probably established, but only one collection.
*Phloeosinus rudis Blandford	FR, NE	1940	3	FR, Hoffman 1942 found many in branches of Thuja japonica, in Var, St. Tropez, in June 1940. Balachowsky cites this. No recent finds. NE, Moraal 2006 and email: apparently locally established, along with Phloeosinus aubei. Kills Thuja occidentalis, Chamaecyparis and Juniperus chinensis. At least rudis is probably established, aubei might be too.
Phloeotribus liminaris (Harris)	IT	2004	2	IT (only), Pennacchio et al. 2004.
Xyleborinus attenuatus (Blandford) ^b^	AT, CH, CZ, DE, ES, NE, PL, SE, RU, SK, UN	1987	5	AT: Holzschuh 1990 (oldest specimen 1986). CH, not in Bovey 1987; Kenis et al. 2005 (“C. Besuchet, pers. comm.”). CZ, Knížek 1988 (1^st^ Eur record). DE, Lohse 1991. ES, Lombardero 1998. NE, Vorst et al. 2008. PL, Lohse 1991. SE, Lindelöw et al. 2006. Western Russia, Ukraine, Nikulina et al. 2007. SK, Knížek 1988.
Xyleborus affinis Eichhoff	AT	2006	3	HU: found in imported Dracaena, no recent records (Merkl Otto, email, Merkl and Tusnadi 1992). IT, regularly in imported Dracaena, e.g. Carrai 1992. AT, “rare”, introd. 2006: AliensAustria 2007 (Holzer 2007, 1 in Malaise trap).
*Xyleborus pfeilii (Ratzeburg)	AT, BG, CH, CA, CZ, DE, ES, FR, HU, IT, PL, SI, SK, UN	1837	5	Infrequently collected, but widespread in Europe and N. Africa. AT and DE, “Gallia”, Eichhoff 1878. BG, 1934 specimens seen by Lombardero (1996). CH, Bovey (1987), not reported since 1898. DE, described from DE by Ratzeburg 1837. ES, Lombardero 1996 did not find, but she cites Kleine 1913 for ES. More widespread in FR (Balachowsky 1949) and AT (Schedl 1980). HU, is in Endrödi 1959. IT, Francardi et al. 2006. PL, is in Nunberg 1954. ES, DE, AT, FR: Reitter 1916, Fauna Germanica. Almost all central and southern European countries, Knížek 2004. Pfeffer 1995: AT, DE, FR, CZ, PL,UN, HU, CA, SI, SK: given the wide distribution of the species, we treat these as records for establishment, though it is not clear if Pfeffer made this distinction.
Xylosandrus crassiusculus (Motschulsky)	IT	2003	2	IT, Pennacchio et al. 2003.
Xylosandrus germanus (Blandford)	AT, BE, CH, CZ, DE, ES, FR, IT, NE	1950	5	AT, Holzschuh 1993 (1^st^ record 1992). BE, Bruge 1995 (1994). CH, Bovey 1987 (1984). CZ, Knížek 2009. DE, Groschke 1953 (1950?). ES, established, López et al. 2007 (2003). FR, Schott 1994 (1984). IT, Stergulc et al. 1999 (1992). NE, Vorst et al. 2008.
Xylosandrus morigerus (Blandford)	AT, CZ, FR, IT, UK	1916	3	UK, FR, AT and CZ (Bohemia), Reitter 1916, as occurring in greenhouses on Dendrobium. FR, greenhouse orchids, Balachowsky 1949. UK (Kew Gardens), Rome, Wien (orchids) in greenhouses, Schedl 1980.

Country abbreviations: **AT** Austria; **BE** Belgium; **BG** Bulgaria; **CA** Croatia, **CH** Switzerland; **CZ** Czech Republic; **DE** Germany; **ES** Spain; **FI** Finland; **FR** France; **GR** Greek; **HU** Hungary; **IT** Italy; **MA** Malta; **NE** Nederland; **PL** Poland; **RU** Russia; **SE** Sweden; **SI** Slovenia; **SK** Slovakia; **UK** United Kingdom; **UN** Ukraine. ^a^ The only Platypodinae; treated as Platypus sulcatus or Platypus mutatus in most earlier literature. ^b^ Treated as Xyleborinus alni (Niijima, 1909) in most literature. *Species not treated as established extra-European aliens in DAISIE.

Nine of our 19 species are not classified as established aliens in DAISIE. We explain their inclusion here briefly. Five on our list are classified by DAISIE as “status unknown”. In two, this is probably due to simple “coding errors”: there is no doubt that widely distributed species as (1) Coccotrypes bodoanum and (2) Hypothenemus eruditus are well-established aliens. That three more restricted species are established aliens is less widely known. (3) Phloeosinus rudis was collected in 1940 from Thuja japonica branches in St. Tropez (Hoffman 1942), suggesting that there was a breeding population in France at that time. The fate of this colony is not known, nor are there any subsequent records of the species from France. However, this species along with Phloeosinus aubei (a Mediterranean species with similar biology) have recently been reported killing ornamental Thuja occidentalis, Chamaecyparis and Juniperus chinensis in the Netherlands (Moraal 2005, 2006). (4) Dryocoetes himalayensis is know only from the Himalayas of India; it has been collected over the past few years from both France and Switzerland (Knížek in press and pers. comm.). (5) Ambrosiophilus atratus was collected at one village in northeastern Italy in 2007 and 2008 in alcohol-baited traps (Faccoli 2008). The beetles clearly had overwintered successfully.

Xyleborus affinis is tentatively included in our list, because of the Malaise trap catch in Austria (Holzer 2007). As long as they are not near piles of imported logs, trap catches are strong evidence of a local, established population, and are now the main source of information on alien species in many regions around the world. This species is also possibly established in nurseries in Italy, where its presence in imported Dracaena stems seems to be constant (Carrai 1992), but it is also possible that these beetles are continuously imported and do not form reproducing populations. If it is indeed established in nurseries, its status in Italy would resemble that of Xylosandrus morigerus in Europe, a species with a long history of reproducing populations in orchids in greenhouses and which also is probably regularly being imported (Table 2).

Two ambrosia beetles on our list but not in DAISIE are only recently discovered: Ambrosiodmus rubricollis (Faccoli et al. 2009), and Monarthrum mali (Kirkendall et al. 2008). Large numbers of the former were collected from a live horse chestnut (Aesculus hippocastaneum) in the botanical gardens of Padua (Apr. 2009), and from peach trees (Prunus persicae) close to Verona (Oct. 2009), both in northeastern Italy. A single Monarthrum mali was trapped in a nature reserve in northeastern Italy in 2007. Given that the species is not often trapped even where it is common and indigenous (in eastern North America), and the remote locality, this species is considered to be established (Kirkendall et al. 2008).

The last species on our list of alien species, Xyleborus pfeilii, is currently considered to be indigenous. This ambrosia beetle is considered rare but found in much of Europe as well as in northern Africa and Turkey (Wood and Bright 1992, Pfeffer 1995); it is also established in both eastern and western North America (Vandenberg et al. 2000, LaBonte et al. 2005). Morphologically, it apparently belongs to the volvulus-perforans group of species (most of which are probably Asian in origin); it is not similar to any of the species of Xyleborus native to Europe. Furthermore, unlike Trypodendron laeve (see below), it shows a clearly disjunct distribution, with what we consider to be the native populations being in southern China, Japan, and Korea (Wood and Bright 1992). We suggest that this species was introduced to Europe from trade with the Far East, and spread so widely that the earliest bark beetle specialists (e.g. Eichhoff 1878) assumed it was part of the native fauna.

Finally, there is one species which we did not include but which may have recently made its first inroads into Europe. The highly aggressive Asian larch bark beetle Ips subelongatus (Motschulsky) has long considered synonymous with the European larch bark beetle Ips cembrae (Heer) (Wood and Bright 1992) but is geographically and genetically distinct and carries different strains of blue-stain fungus (Stauffer et al. 2001). The two can be distinguished morphologically by specialists familiar with both species. Both species breed normally in larch (Larix), but are occasionally found breeding in alternative hosts. Ips subelongatus was intercepted in Finland in logs from Siberia and in Estonia in timber from Russia (Voolma et al. 2004). Larch bark beetles were taken from spruces around St. Petersburg and more recently have been collected from pines in the Murmansk province (Voolma et al. 2004), which is outside the natural range of larch. Given the regional trade patterns, it is possible that these are Ips subelongatus, but species identity has not been confirmed by taxonomists or DNA data.

Two species are listed by DAISIE (2009a) as established aliens which we classify differently. The record for Xyleborus perforans seems to be based on a one-time interception from imported logs, in Poland (Wojciech Solarz, pers. comm.). There is no evidence for Poland or elsewhere that this widespread tropical ambrosia beetle reproduces anywhere in Europe. Trypodendron laeve Eggers, on the other hand, we propose is actually native to Europe. This spruce-breeding ambrosia beetle is treated by DAISIE, NOBANIS, and in the recent forestry literature (e.g. Kenis 2005) as an alien species. The perception that it is exotic presumably arose because it was first described from Japan, and subsequently only known in the West from Norway, Sweden, and Austria (Pfeffer 1995). However, Trypodendron laeve is apparently rare; it remained unknown to science until 1939, when Eggers described it based on a five specimens from Japan, and only seven years later when Strand unknowingly described as Trypodendron piceum the same species from a single collection from near Oslo, Norway (Eggers 1939, Strand 1946). The accumulation of collection data reflected in DAISIE and Fauna Europaea (and in Knížek’s upcoming Palaearctic Scolytinae catalog) reveals a species which has now been found throughout Europe and across Asia to Japan, much like more common conifer forest bark beetles such as Ips typographus or Tomicus piniperda. We see no reason to continue to consider this species to be alien to Europe.

The written list of aliens (DAISIE 2009b) includes 20 Scolytinae and 3 Platypodinae. Unlike the web version, these records do not specify status, so all records are presumably considered to be established species, and treated such in DAISIE’s many analyses of terrestrial invertebrate or insect invasions. Of these 23 species, 13 are on this list as established aliens to Europe; one (Phloeotribus caucasicus Reitter) is a spreading European species; seven are interceptions (no evidence of breeding populations in Europe); and one we argue here is a native species (Trypodendron laeve). The net result is that where DAISIE (2009b) would include 23 species of Scolytinae and Platypodinae in analyses of established alien insects in Europe, we propose there are ca 25% fewer (19, only 13 of which are, in fact, listed by DAISIE as established aliens).

#### The genera present.

Clearly, a wide variety of bark beetles are capable being transported to Europe, and there is a surprisingly high diversity which have succeeded in colonizing the continent: the 18 alien species comprise 16 different genera (15 of Scolytinae, 1 of Platypodinae), of which only five are present in the native fauna. Only two genera, Xylosandrus and Xyleborus, are represented by more than one exotic species; the Xyleborini (these two, plus Ambrosiodmus, Ambrosiophilus, Cyclorhipidion, and Xyleborinus) make up half of all adventive species.

### When did they arrive?

**Figure 1.**
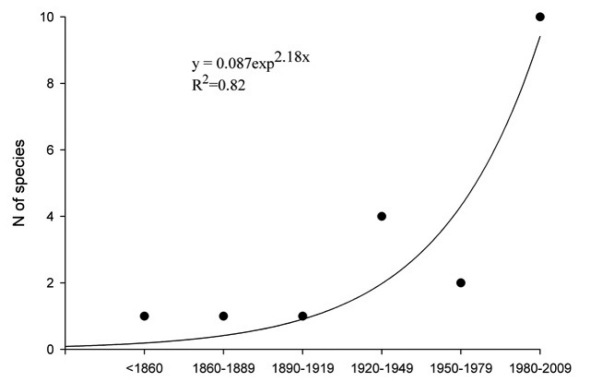
The accelerating rate of discovery of introduced Scolytinae and Platypodinae in Europe, shown as numbers of new species found in each 30-year period (data from Table 2).

The precise date of arrival in Europe is not known for most species because the introductions of bark beetles (and of most animals) are unintentional, and up to several decades can go by before newly established exotics – especially those which are not pests – are noticed (Kenis et al. 2007, Mattson et al. 2007, Roques et al. 2009). The first reference to the presence of an alien scolytine in Europe is the description almost two centuries ago of the east Asian ambrosia beetle Xyleborus pfeilii as a European species (Ratzeburg 1837), followed by reports of the topical seed beetle Coccotrypes dactyliperda in Italy (Targioni-Tozzetti 1884). Only nine more new alien species were found in the next 115 years, though at least one of these (the tiny, highly polyphagous but harmless Hypothenemus eruditus firstrecorded by Ragusa in 1924) could well have been present much longer. The successful establishment of exotics seems now be accelerating (Fig. 1), despite greater international awareness of the dangers posed by wood packing materials (FAO 2002) and stricter regulation of plant trade: fully 8/19 aliens have been reported in the last decade (Table 2). The establishment rate in Europe of new alien species of insects (and of terrestrial invertebrates generally) has increased markedly in the last thirty years (Hulme et al. 2009).

### How were they transported?

Many wood-boring insects, particularly scolytine and platypodine beetles, are transported between continents. While the majority of introductions of alien insects to Europe is via trade in ornamental plants (Kenis et al. 2007, Roques et al. 2009), bark beetles mainly travel in wood and in wooden packing materials such as crating, dunnage and pallets (Haack 2001, Allen and Humble 2002, Colunga-Garcia et al. 2009, Haack and Petrice 2009). Only a few are likely to be transported in plants or plant parts. The cut stems of Dracaena which are shipped to Europe from Central America frequently are infested with tropical Xyleborus species, the seeds and nuts with Coccotrypes, Dactylotrypes, and Hypothenemus, and the orchids with Xylosandrus morigerus; Hypocryphalus scabricollis probably entered Malta with exotic Ficus trees from southern Asia (Mifsud and Knížek 2009).

### Biology of Europe’s alien bark beetles

Whether or not alien insects succeed in establishing breeding populations depends on a number of factors, including suitability of local climate and hosts, appropriate phenology, and the effects of potential competitors and natural enemies. Immigrants which are host generalists or which use host species which are abundant and widespread where they have arrived should have a good chance of establishing permanent populations, given appropriate climatic conditions.

#### Niche breadth.

The vast majority of bark beetles (particularly phloeophagous species) are monophagous, breeding in one genus of host plants, or oligophagous, breeding in one family of host plants (Beaver 1979, Kirkendall 1983). These breed in one species of woody plant, several species in one genus, or in several related genera of hosts. Strikingly, all but four of the established aliens of mainland Europe are polyphagous (breeding in several to many families of woody plants). Two-thirds of the established alien Scolytinae and Platypodinae are ambrosia beetles, a much higher proportion than would be found in the source faunas of Asia or North America (Kirkendall 1993). Ambrosia beetles are most often polyphagous (Beaver 1979, Kirkendall 1983), and lack of host specificity is considered to be a major reason why they are so successful as invaders (Atkinson et al. 1990, Kirkendall et al. 2008). Of those species with more restricted diets, two breed in palm seeds, an abundant resource all around the Mediterranean, one in Fagaceae (a dominant family in much of Europe), and one in widely planted fruit trees (Table 3).

**Table 3.** Source and biology of alien bark beetles of Europe. Data from sources in Table 2, Wood (1982), Kirkendall (1983) and Wood and Bright (1992).

Species	Native to	Additional distribution	Zone	Feeds/Breeds	Host use
Ambrosiodmus rubricollis	east Asia	eastern North America, Australia	T	Xm/inbreeding	Polyphagous, broadleaf trees
Ambrosiophilus atratus	east Asia	North America	T	Xm/inbreeding	Polyphagous, broadleaf trees
Coccotrypes dactyliperda	? (Old World)	globally distributed, tropics & subtropics	M	Spm/inbreeding	Polyphagous, mainly palm seeds in Europe
Cyclorhipidion bodoanum	north Asia	North America	T	Xm/inbreeding	Oligophagous, Fagaceae
Dactylotrypes longicollis	Canary Islands	Madeira, North Africa	M	Spm/outbreeding	Oligaphagous, palm seeds
Dryocoetes himalayensis	India		T	Phl/outbreeding	Polyphagous, Juglans regia, Pyrus lanata
Gnathotrichus materiarius	eastern N. America		T	Xm/outbreeding	Polyphagous, conifers
Hypocryphalus scabricollis	east Asia		M	Phl/outbreeding	Polyphagous, broadleaf trees
Hypothenemus eruditus	American tropics?	globally distributed, tropics & subtropics	M	Phl/inbreeding	Polyphagous
Megaplatypus mutatus	South America		M	Xm/outbreeding	Polyphagous, broadleaf trees
Monarthrum mali	eastern N. America		T	Xm/outbreeding	Polyphagous, broadleaf trees
Phloeosinus rudis	east Asia		T	Phl/outbreeding	Oligophagous, Cupressaceae
Phloeotribus liminaris	eastern US		M	Phl/outbreeding	Monophagous, Prunus
Xyleborinus attenuatus	east Asia	North America	T	Xm/inbreeding	Polyphagous, broadleaf trees
Xyleborus affinis	Neotropics?	globally distributed, tropics & subtropics	M	Xm/inbreeding	Polyphagous
Xyleborus pfeilii	east Asia	North America	T,M	Xm/inbreeding	Polyphagous, broadleaf trees in Europe*
Xylosandrus crassiusculus	tropical & subtropical Asia	globally distributed, tropics & subtropics	M	Xm/inbreeding	Polyphagous
Xylosandrus germanus	east Asia	North America	T	Xm/inbreeding	Polyphagous
Xylosandrus morigerus	Asian tropics?	globally distributed, tropics	gh	Xm/inbreeding	Polyphagous; in Europe, greenhouse orchids

Additional distribution: other foreign regions in which a species is now established. Zone: **T**, temperate zone of Europe; **M**, Mediterranean zone; **B**, boreal zone; **gh**, greenhouse populations. Feeds: **Xm**, xylomycetophagous (ambrosia beetle); **Phl**, phloeophagous, breeding in bark; **Spm**, spermatophagous, breeding in seeds (terminology from Wood 1982). * Xyleborus pfeilii is highly polyphagous in conifers and broadleaf trees in Japan ( Mizuno and Kajimura 2002) though the few host records in Europe are from Alnus and Betula (e.g. Balachowsky 1949).

#### Importance of reproductive system.

Particularly important to recently established, small populations are Allee effects, the acute demographic, ecological and genetic problems posed by low densities (Lande 1988, Courchamp et al. 2008). Single small populations are always at risk of extinction from random local disasters, and if they arose from large outbreeding populations they will usually suffer from inbreeding depression. Mate location can also lower the reproductive rate of small populations. Species which regularly mate by brother-sister mating, however, circumvent many of these problems: mating takes place among siblings, before dispersal, and regular inbreeders presumably suffer much less from inbreeding depression than do outbreeders (Jordal et al. 2001, Frankham et al. 2004, Kirkendall and Jordal 2006). Eleven (58%) of the immigrant species are inbreeders (Table 3), which is roughly twice as high as the proportion of the European bark beetle fauna which inbreeds (Kirkendall 1993). Inbreeding is also clearly over-represented in adventive bark beetles in North American (Wood 1977, Atkinson et al. 1990, Haack 2001). Of the 50 exotic species established in North America by the year 2000, 37 (74%) are inbreeders (Haack 2001). And, of the 62 North and Central American species recorded as introduced to or exported from foreign countries (Wood 1977), 45 (73%) inbreed. Supporting the importance of inbreeding in colonization, it should be noted that islands almost always have much higher proportions of inbreeding species than their source populations (Kirkendall 1993, Jordal et al. 2001).

Both inbreeding and polyphagy should favor invasiveness. Interestingly, 10/15 polyphagous species are inbreeders, and 10/11 inbreeders are polyphagous (Table 4).

**Table 4.** The relationship between feeding habits and reproductive systems, for alien Scolytinae and Platypodinae established in Europe. Data from Table 3.

Reproduction type	Polyphagous	Not polyphagous
Inbreeding	10	1
Outbreeding	5	3

### Biogeography: Where are alien species established, and where did they come from?

#### Climatic zones of Europe.

Though smaller in area, the Mediterranean zone is disproportionately rich in alien bark beetles (Table 3). Mediterranean ecosystems are particularly rich in biodiversity (Underwood et al. 2009) and have milder winters than elsewhere in Europe, two factors which might favor the establishment of newly arrived species. Only the oldest established exotic, Xyleborus pfeilii, is currently established in two different zones (temperate and Mediterranean). In Europe, as far as is known, the tropical ambrosia beetle Xylosandrus morigerus is restricted to greenhouses where it is a pest of orchids.

#### Country records.

**Figure 2.**
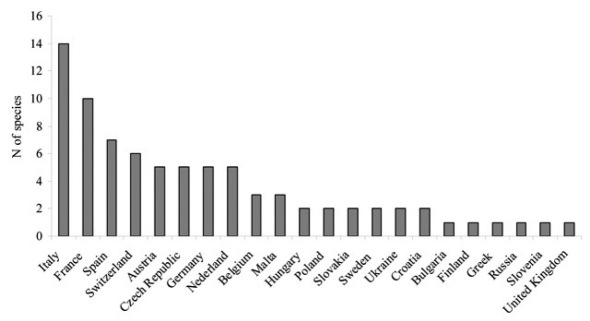
The numbers of alien bark beetles and pinhole borers per European country (data from Table 2).

Although 22 European countries recorded exotic species, large differences exist among the numbers of alien insects recorded per country (Fig. 2). Italy, France and Spain have the largest numbers of alien species (14, 10 and 7, respectively); for the former two countries, this corresponds to about 10% of their national bark beetle fauna (Balachowsky 1949, Abbazzi et al. 1995). Over half of these countries recorded only one or two alien scolytines and platypodines.

The great differences among countries could be due to several reasons. The number of alien insects is positively correlated with country surface area (Roques et al., 2009). Furthermore bark beetles show a latitudinal gradient in species richness: the number of alien scolytines and platypodines generally decreases with the increasing latitude (Kirkendall 1993), probably because of harsher winters and reduced host diversity. Besides having favorable temperatures, the southern countries (Italy, France and Spain) also have a wide variety of ecosystems, ranging from Mediterranean to mountain and alpine, and of climate regimes, leading to high diversity of woody plants and of ecological conditions.

While some of the differences between countries are real – Sweden does have fewer invasives than Switzerland – others are due to under-reporting at the country level; certainly, many of the differences among countries are due to differences in collecting effort and to the presence (or absence) of researchers with a special interest for Scolytinae and Platypodinae. Many sub-Scandinavia European countries are represented by zero or few records of alien bark beetle species but do have the requisite habitats. We found it particularly difficult to find detailed information on the bark beetle faunas of Portugal, eastern Europe, the Balkan countries, and countries of the eastern Mediterranean. Alien species doubtlessly can be found in these areas. The true ranges of alien bark beetles will not be known as long as there remain such gaps in our knowledge.

Unfortunately, here, too, the publicly available information on alien species in Europe is largely incorrect. Only for those recent arrivals established only in Italy are the country records in DAISIE accurate. Even species which have been established for over half a century and are well studied are not correctly reported in DAISIE: for both Gnathotrichus materiarius and Xylosandrus germanus, we can document at least three country occurrences missing from DAISIE.

The data in Fauna Europaea are similarly flawed. Three species are missing from the database, four country occurences (for three species) cannot be verified, and country records are incomplete for most alien species, including for Gnathotrichus materiarius (2 missing) and Xylosandrus germanus(3).

#### Where are the exotics from?

By far, the vast majority of recent interceptions of non-indigenous plant pests in European countries are from Asia or from Europe, with an order of magnitude fewer interceptions originating in North America (Roques and Auger-Rozenberg 2006, Mattson et al. 2007). Established alien bark beetles are not as skewed with respect to geographic origin: the majority (12/19) are known or suspected to be native to Asia, but fully six are from the Americas. Of course, geographic origin and origin of immigrant populations can be two different things: five species are globally distributed, five Asian species are also established in North America, and the Canary Island endemic is well established on Madeira and in Morocco (Kirkendall, unpublished data). In most cases, whether Asian species were introduced from Asia or from invasive populations in the New World cannot easily be determined without detailed DNA studies.

The tropical affinities of one-third of the species (Table 3) might come as a surprise to some. However, all but Xylosandrus morigerus range into temperate climes – and that one exception is only found in greenhouses, in Europe.

### Taxonomy and invasives

Increasingly, governments at all levels realize the severity of threat posed by alien insects, and national and international programs have been set in motion throughout the world to address the problem (e.g. McNeely et al. 2001, DAISIE 2009a). However, though often not fully appreciated, correct identification of newly encountered exotic species bedevils many such efforts. As an example, the correct identification of the now well-established ambrosia beetle Cyclorhipidion bodoanum took over three decades and confounded bark beetle specialists on two continents simultaneously. In 1975, Steve Wood described Xyleborus californicus from specimens collected in northern California in 1944 (Wood 1975); he stated that this species was almost certainly exotic and probably from South America or southeastern Asia. The latter suggestion was supported when a specimen of Xyleborus californicus from China was intercepted in Vancouver (Vandenberg et al. 2000). That Xyleborus californicus might actually be Cyclorhipidion bodoanum was suggested subsequently(M. Mandelshtam pers. comm., quoted in Rabaglia et al. 2006); the synonymy will be published by Knížek (pers. comm.) and has been independently verified by the senior author. Meanwhile, in Europe, an invasion by the same ambrosia beetle was initially misidentified as being Xyleborus peregrinus Eggers 1944 (which species actually is a synonym of Xyleborinus saxesenii); this later was corrected to Xyleborinus punctulatus Kurentzov, which name was later shown to be a junior synonym of Xyleborinus bodoanus (Mandelshtam 2001). That Xyleborinus bodoanus is actually a Cyclorhipidion was recognized recently (Bussler 2006). Only now, over a half century since having invaded two continents, does this oriental species appear to be conclusively identified. As illustrated by this example, even specialists are often stymied when introduced species are from Asia, for which we generally lack the most basic tools for species-level identification (keys and high quality illustrations), and for where only a few working bark beetle taxonomists have access to representative material.

Taxonomy plays a fundamental but often underappreciated or overlooked role in strategies for monitoring, intercepting, and managing both exotic and indigenous organisms, including wood borers. Phytosanitary efforts to monitor or control new invasive species will fail without correct taxonomic and biogeographic information (and the latter is dependent on the former). Cryptic species often differ in key elements of their biology, such as in phenology, host preferences, pheromone behavior, susceptibility to natural enemies (including diseases), and in the species or strains of microorganisms which they carry with them. When such differences exist between look-alike species, control measures will often be ineffective if the species is misidentified. For example, similar appearing species may originate from different regions; incorrect identification in such an instance could lead to fruitless searches for key biological control agents. Occasionally, taxonomists themselves have overlooked minute morphological differences between sister species, but more often the incorrect identifications are by nonspecialists relying on published databases, keys, and illustrations rather than on consultation with taxonomic experts (Knížek 2007). On the other hand, experts are reluctant (or unable) to invest time in “routine identifications” involving thousands or tens of thousands of specimens of abundant pest species.

The taxonomic impediment is often three-fold: difficult access to taxonomic specialists; poor taxonomic knowledge of the group involved; lack of user-friendly keys and illustrations. Taxonomic specialists are few and overworked; quarantine agencies, foresters and other instances must compete with taxonomists’ own research projects (and more and more with specimen-rich biodiversity surveys). Taxonomic knowledge can be inadequate in several ways: many genera of wood-boring insects (including scolytines and platypodines) have not been recently revised (some, never so); for some regions of the world, the wood-boring fauna is poorly known; and for some species groups which are highly successful as colonists, species-boundaries and proper nomenclature are inadequately understood. Finally, even where the wood-borers are fairly well known and keys do exist (e.g. Central America), for many genera the keys can only be used by specialists with access to reference material; illustrations sufficient for species-level identification (drawings or high-resolution photographs) exist only for a very limited number of species groups or genera.

A way out of this impasse is two-fold: use of adequate photographic documentation of subtle morphological differences, especially when coupled with expert intelligence software for developing illustration-rich keys; and the development of inexpensive molecular methods (fragment profile- or sequence-based) for separating species difficult to identify by morphology (DNA barcoding). Fortunately, tools for both are becoming increasingly well known and more widely accessible, as are possibilities to publish new finds rapidly via highly accessible electronic journals. Consequently, we are already seeing that new discoveries are being documented, identified, illustrated and published much more rapidly.

In the future there will be more and more Asian wood-borers colonizing Europe and North America. Currently there are no modern resources for identifying bark beetles from Asia, the Orient, or Oceania. What is needed is the methodical, thorough monographic work which Steve Wood was so good at, preferably including DNA sequencing. Until we have monographs for China, Southeast Asia, and Oceania – and the young taxonomic talents capable of applying them –many future immigrants will long remain enigmas.
